# Personalized app-based coaching for improving physical activity in heart failure with preserved ejection fraction patients compared with standard care: rationale and design of the MyoMobile Study

**DOI:** 10.1093/ehjdh/ztae096

**Published:** 2025-01-30

**Authors:** Silav Zeid, Jürgen H Prochaska, Alexander Schuch, Sven Oliver Tröbs, Andreas Schulz, Thomas Münzel, Tanja Pies, Wilfried Dinh, Matthias Michal, Perikles Simon, Philipp Sebastian Wild

**Affiliations:** Preventive Cardiology and Preventive Medicine, Department of Cardiology, University Medical Center of the Johannes Gutenberg University Mainz, Langenbeckstraße 1, 55131 Mainz, Germany; German Center for Cardiovascular Research (DZHK), Partner Site Rhine-Main, Mainz, Langenbeckstraße 1, 55131 Mainz, Germany; Preventive Cardiology and Preventive Medicine, Department of Cardiology, University Medical Center of the Johannes Gutenberg University Mainz, Langenbeckstraße 1, 55131 Mainz, Germany; German Center for Cardiovascular Research (DZHK), Partner Site Rhine-Main, Mainz, Langenbeckstraße 1, 55131 Mainz, Germany; Clinical Epidemiology and Systems Medicine, Center for Thrombosis and Hemostasis (CTH), University Medical Center of the Johannes Gutenberg University Mainz, Langenbeckstraße 1, 55131 Mainz, Germany; Boehringer Ingelheim, Ingelheim am Rhein, Binger Str. 173, 55218 Ingelheim am Rhein, Germany; Preventive Cardiology and Preventive Medicine, Department of Cardiology, University Medical Center of the Johannes Gutenberg University Mainz, Langenbeckstraße 1, 55131 Mainz, Germany; German Center for Cardiovascular Research (DZHK), Partner Site Rhine-Main, Mainz, Langenbeckstraße 1, 55131 Mainz, Germany; Preventive Cardiology and Preventive Medicine, Department of Cardiology, University Medical Center of the Johannes Gutenberg University Mainz, Langenbeckstraße 1, 55131 Mainz, Germany; German Center for Cardiovascular Research (DZHK), Partner Site Rhine-Main, Mainz, Langenbeckstraße 1, 55131 Mainz, Germany; Boehringer Ingelheim, Ingelheim am Rhein, Binger Str. 173, 55218 Ingelheim am Rhein, Germany; Preventive Cardiology and Preventive Medicine, Department of Cardiology, University Medical Center of the Johannes Gutenberg University Mainz, Langenbeckstraße 1, 55131 Mainz, Germany; German Center for Cardiovascular Research (DZHK), Partner Site Rhine-Main, Mainz, Langenbeckstraße 1, 55131 Mainz, Germany; Department of Cardiology — Cardiology I, University Medical Center of the Johannes Gutenberg University Mainz, Langenbeckstraße 1, 55131 Mainz, Germany; Bayer AG, Friedrich-Ebert-Straße 217/333, 42117 Wuppertal, Germany; Bayer AG, Friedrich-Ebert-Straße 217/333, 42117 Wuppertal, Germany; Institute for Cardiovascular Research, University of Witten/Herdecke, Alfred-Herrhausen-Straße 50, 58455 Witten, Germany; Department of Cardiology, HELIOS Clinic Wuppertal, Arrenberger Str. 20, 42117 Wuppertal, Germany; German Center for Cardiovascular Research (DZHK), Partner Site Rhine-Main, Mainz, Langenbeckstraße 1, 55131 Mainz, Germany; Department of Psychosomatic Medicine and Psychotherapy, University Medical Center of the Johannes Gutenberg-University Mainz, Langenbeckstraße 1, 55131 Mainz, Germany; Department of Sports Medicine, Rehabilitation and Disease Prevention, Faculty of Social Science, Media and Sport, Johannes Gutenberg-University Mainz, Albert-Schweitzer-Straße 22, 55128 Mainz, Germany; Preventive Cardiology and Preventive Medicine, Department of Cardiology, University Medical Center of the Johannes Gutenberg University Mainz, Langenbeckstraße 1, 55131 Mainz, Germany; German Center for Cardiovascular Research (DZHK), Partner Site Rhine-Main, Mainz, Langenbeckstraße 1, 55131 Mainz, Germany; Clinical Epidemiology and Systems Medicine, Center for Thrombosis and Hemostasis (CTH), University Medical Center of the Johannes Gutenberg University Mainz, Langenbeckstraße 1, 55131 Mainz, Germany; Systems Medicine Group, Institute of Molecular Biology (IMB), Ackermannweg 4, 55128 Mainz, Germany

**Keywords:** Clinical trial, Heart failure with preserved ejection fraction (HFpEF), Physical activity, Digital mobility outcome, Mobile app

## Abstract

**Aims:**

Patients suffering from heart failure with preserved ejection fraction (HFpEF) often exhibit a sedentary lifestyle, contributing to the worsening of their condition. Although there is an inverse relationship between physical activity (PA) and adverse cardiovascular outcomes, the implementation of Class Ia PA guidelines is hindered by low participation in supervised and structured programmes, which are not suitable for a diverse population of HFpEF patients. The MyoMobile study has been designed to assess the effect of a 12-week, app-based coaching programme on promoting PA in patients with HFpEF.

**Methods and results:**

The MyoMobile study was a single-centre, randomized, controlled three-armed parallel group clinical trial with prospective data collection to investigate the effect of a personalized mobile app health intervention compared with usual care on PA levels in patients with HFpEF. Major inclusion criteria were age ≥ 45 years, a diagnosis of HFpEF, LVEF > 40%, and current HF symptoms (NYHA Class I–III). Major exclusion criteria included acute decompensated HF, non-ambulatory status, recent acute coronary syndrome or cardiac surgery, alternative diagnoses for HF symptoms, active cancer treatment, and physical or medical conditions affecting mobility. Participants were recruited from hospitals, general practices, and practices specialized in internal medicine and cardiology in the Rhine-Main area, Germany. Participants underwent an objective 7-day PA measurement with a 3D accelerometer (Dynaport, McRoberts) at screening and after the 12-week intervention period. Following the screening, eligible participants were randomized into one of three groups: standard care (PA consulting), the intervention arm with app-based PA tracking and coaching, or the intervention arm with tracking but without coaching. The primary efficacy endpoint was the change in average daily step count between the average step count at baseline and at the end of the intervention, comparing standard care to a 12-week app-based PA coaching intervention.

**Conclusion:**

Exercise intolerance is a primary symptom in HFpEF patients, leading to poor quality of life and HF-related adverse outcomes due to physical inactivity. The MyoMobile study was designed to investigate the use of app-based coaching to improve PA in patients with HFpEF with a personalized, home-based intervention, focusing on simple step counts for flexibility and ease of integration into daily routines.

**Clinical trial registration:**

URL: https://clinicaltrials.gov/ct2/show/NCT04940312.

**Unique identifier:**

NCT04940312.

## Graphical Abstract

**Graphical Abstract:**
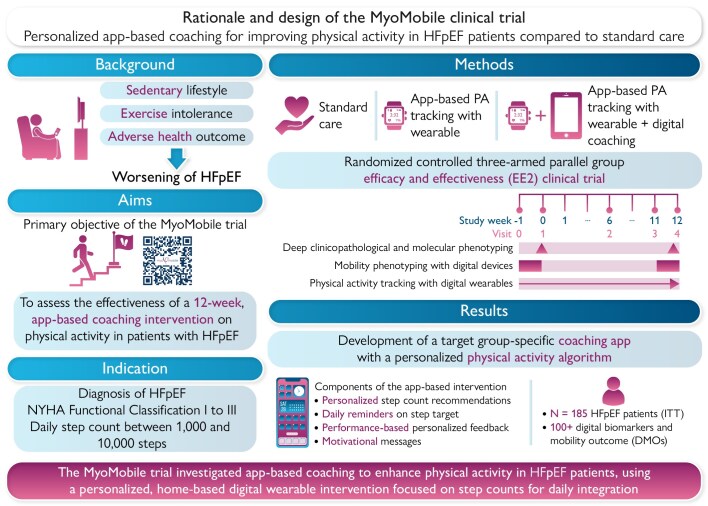

## Introduction

Heart failure (HF) is a heterogeneous, complex syndrome that is a major public health issue in developed countries. The prevalence of HF has increased over the past decades due to a number of factors, including population ageing and consequently higher prevalence of predisposing risk factors.^1^ Approximately half of the HF population is burdened with HF with preserved ejection fraction (HFpEF), for which there are currently limited effective pharmacological therapies.^2^

Exercise intolerance and impaired functional capacity due to physical inactivity are among the cardinal symptoms experienced by HFpEF patients in daily life, underlining the healthcare needs for this patient population.^3,4^ While physical activity refers to any movement that increases energy expenditure, cardiac rehabilitation encompasses a more structured approach including supervised exercise training alongside additional components like nutritional and psychological counselling and education for heart-healthy living. The distinction between these two approaches to lifestyle enhancement is essential, as each addresses distinct facets of patient care. Findings from recent studies suggest that the level of physical activity in patients with HF might be modifiable with exercise training.^3^ In patients with HFrEF, it was demonstrated that increased physical activity is positively associated with improvement in clinical status and patient-reported outcomes, whereas irregular or absent exercise is associated with worsening clinical status and quality of life.^3,5^

Guidelines from the European Society of Cardiology recommend at least 30 min of moderate-intensity activity for ≥5 days/week.^6^ Unfortunately, exercise recommendations are poorly followed in daily clinical practice, and even patients enrolled in supervised exercise programmes are rarely compliant.^7,8^ To overcome these limitations and increase adherence to lifestyle interventions, an app-based, personalized lifestyle intervention could be a promising alternative because it would incorporate a familiar and convenient flexible intervention based on a simple step count, making it easier to implement in the normal daily routine.^9^ Such an intervention focuses on encouraging daily physical activity, in particular step count, which could be more easily incorporated into the daily routine without the infrastructure or supervision required by formal rehabilitation programmes.

Multiple clinical trials have demonstrated that the use of wearables with digital applications, such as pedometers and fitness wristbands, improves the level of physical activity in individuals with chronic obstructive pulmonary disease^10^ and older people.^11^ However, limited data are available assessing the feasibility, validity, and effectiveness of novel, app-based approaches to personalized exercise training programmes for patients with HFpEF.^12,13^

The MyoMobile study was designed as a randomized parallel-arm clinical trial to investigate the effects of a personalized coaching app on physical activity and biomedical outcomes with a focus on the cardiovascular system in patients with HFpEF.

## Methods

### Study design

The MyoMobile study was an investigator-initiated, single-centre, randomized, controlled, three-armed parallel group clinical trial with prospective data collection to investigate the effect of a personalized app-based coaching intervention on the physical activity levels in patients with HFpEF during the intervention period of 12 weeks compared to usual care and physical activity tracking alone. The MyoMobile study was an efficacy and effectiveness too (EE2) trial, i.e. simultaneously addressing efficacy and effectiveness in a so-called EE2 trial. This dual-focused approach, as detailed by Selker *et al*.,^14^ allows for a comprehensive evaluation of how the personalized, app-based coaching intervention performs under controlled conditions (efficacy) and in real-world settings (effectiveness), offering valuable insights into its applicability for patients with HFpEF.

**Figure 1:**
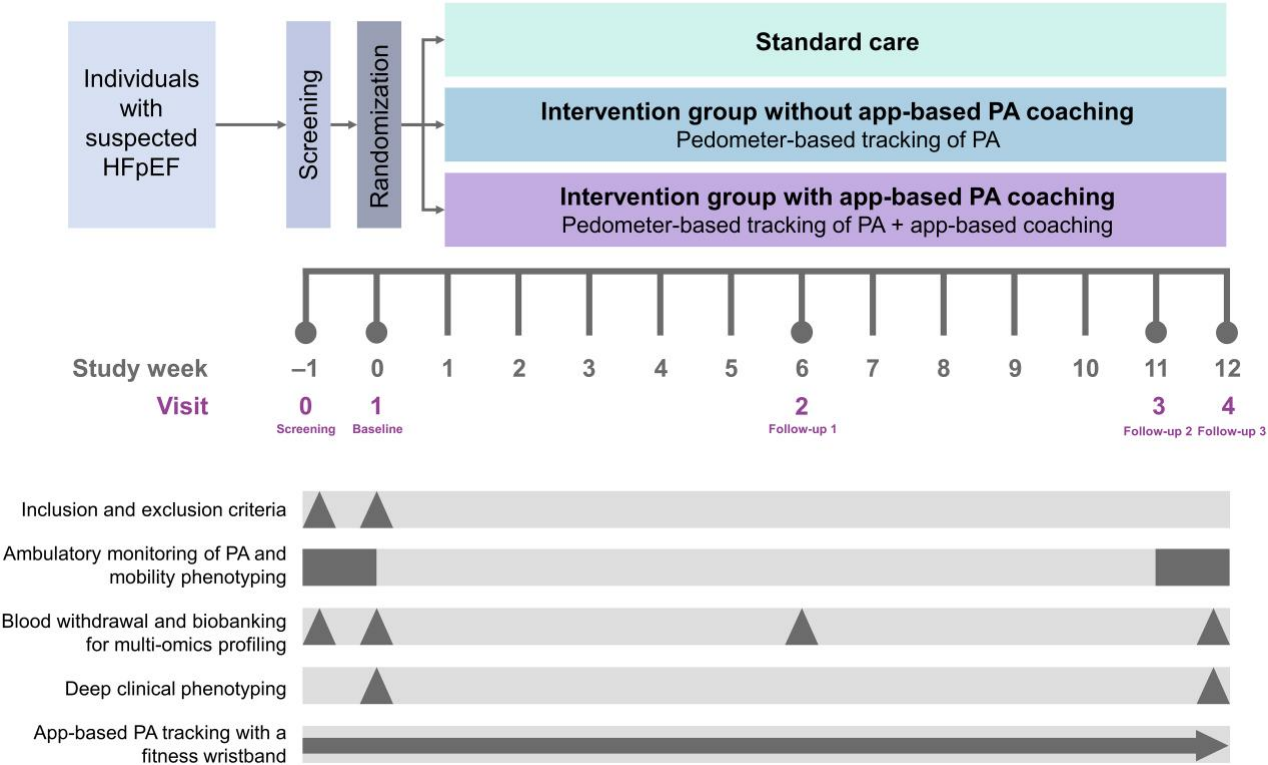
Study design of the MyoMobile study from screening to the end of the intervention, i.e. 12-week follow-up. Triangle, points towards the visit; rectangle, measured for 7 days; arrow, continuously measured.

The study structure comprised a screening phase of 7 days prior to the 12-week intervention period, and each participant was required to complete five visits during the entire study period. The screening (Visit 0) comprised 7 days before the baseline visit (Visit 1), which corresponded to Day 0. Visit 2 was in Week 6 after baseline and Visit 3 in Week 11 after baseline with a time window of −7 to +14 days. Visit 4 was conducted in Week 12 after baseline with a time window of −10 to +14 days. *Figure 1* displays the study flow.

### Study objectives

The primary objective of the MyoMobile study was to investigate the impact of a 12-week personalized activity coaching programme utilizing a smartphone-based app on the average daily step count, as surrogate measure of physical activity in patients with HFpEF.

The first secondary objective was to compare the effect of the aforementioned 12-week personalized, app-based activity coaching intervention on daily step count to the effect of physical activity tracking for 12 weeks without the app-based activity coaching. This second intervention arm with physical activity tracking without concomitant activity coaching was included to explore the potential effect of physical activity tracking alone without personalized coaching on daily step count. Other secondary objectives included assessing the effects of the 12-week personalized app-based activity coaching intervention on cardiac function and structure, quality of life, heart rate variability, cardiopulmonary exercise capacity, autonomic function, and neurohumoral activation compared with standard care and compared with 12 weeks of app-based physical activity tracking without activity coaching. Supplementary material online, *Table S1* provides a detailed overview of the secondary objectives.

The tertiary objectives include the assessment of the effects of the 12-week personalized app-based activity coaching intervention compared to 6-week intervention with and without app-based activity coaching on biomarkers of cardiovascular disease, autonomic function, vascular and endothelial function, daytime activities, and mobility (see Supplementary material online, *Table S2* for a full overview). Besides the extensive exploration of biomarkers, the mobile devices used in this trial will be evaluated with regard to technical implementation, wearability, data assessment, and agreement of recorded ambulatory data with established measurement methods of exercise capacity (e.g. correlation of ambulatory accelerometry data with results of the 6 min walk test and spiroergometry). Additionally, the MyoMobile trial will establish a biobank that will allow researchers to systematically explore biomedical hypotheses in the context of HFpEF and physical activity.

### Regulatory aspects, ethics, and data safety

The local data protection office and ethics committee approved the study protocol and related documents before trial initiation (processing number: 2020-15236). The local ethics committee must approve significant changes to the study protocol. The University Medical Center Mainz of the Johannes Gutenberg University Mainz conducted the trial as study sponsor with financial support from Bayer AG. Bayer AG contributed to the study design and interpretation of the results, but did not conduct the trial or influence the publication of the results. The trial met the recommendations and requirements of the Declaration of Helsinki^15^ and recommendations for good clinical practice as stated by the European Medicines Agency. The trial was registered at http://clinicaltrials.gov with the identifier NCT04940312.

### Study population, patient recruitment, and randomization

The study population included patients aged ≥45 years with a diagnosis of HFpEF with a left ventricular ejection fraction (LVEF) > 40% and New York Heart Association (NYHA) functional Class I to III, who were receiving standard therapy according to current HF guidelines.^16^  *Table 1* provides a detailed description of all inclusion and exclusion criteria. The MyoMobile trial was planned to begin with the first-patient-first-visit in October 2020 (during the second COVID-19 wave) and to last approximately 18 months, concluding with the last-patient-last-visit in March 2022. Participants were recruited from hospitals, general practices, practices for internal medicine and cardiology practices, and cardiac rehabilitation centres in the Rhine-Main area of Germany. In addition, an advertisement for the MyoMobile trial was placed in local newspapers.

**Table 1:** Eligibility criteria

Inclusion criteria	Exclusion criteria
Age ≥ 45 years	Acute decompensated HF requiring augmented therapy with diuretic agents, vasodilator agents, and/or inotropic drugs
Diagnosis of HFpEF	Participants who are non-ambulatory managed or use mobility assistive devices such as motorized devices or wheelchairs
LVEF > 40% by any imaging modality at screening within 4 months prior to study entry	Acute coronary syndrome (including myocardial infarction), cardiac surgery, other major cardiovascular surgery, or urgent PCI within 3 months prior to Visit 1 or an elective PCI within 30 days after study enrolment
Current HF symptoms as defined as presence of dyspnoea according to NYHA functional Class I to III at screening visit	Probable alternative diagnoses that in the opinion of the investigator account for the patient’s HF symptoms (i.e. dyspnoea and fatigue)
Stable HF treatment for at least 4 weeks prior to screening	Current chemotherapy and/or radiation therapy for treatment of active cancer
At least one of the following criteria need to be fulfilled:	Medical or psychological conditions that would jeopardize an adequate and orderly conduct or completion of the study
NT-proBNP ≥ 300pg/mL	Participants with physical activity impairment primarily due to conditions other than HF such as the following:
Hospitalization for HF within the past 12 months	Participants unwilling or unable to wear or to operate study measurement devices for the phases required
Symptom(s) of HF requiring treatment with diuretic(s) for at least 30 days prior to screening visit	Exertional angina, foot ulcer (e.g. diabetic foot syndrome), and/or prosthetic limbs
Wearing time of the physical activity monitor for at least 4 days during the baseline assessment	Inflammatory disease or degenerative joint disease or peripheral vascular disease
Average daily step count during baseline assessment ≥ 1000 steps per day and <10 000 steps per day	Neurologic disease affecting activity or mobility (e.g. peripheral neuropathy)

Individuals meeting all the inclusion criteria and none of the exclusion criteria at Visit 0 (i.e. screening) were considered for participation in the study. Individuals presenting with at least one of the following exclusion criteria at Visit 0 or at Visit 1 (screening and baseline, respectively) were not enrolled in the study. HF, heart failure; HFpEF, HF with preserved ejection fraction; NT-proBNP, N-terminal pro b-type natriuretic peptide; PCI, percutaneous coronary intervention.

Participants were examined at the MyoMobile study platform located at the Preventive Cardiology and Preventive Medicine, Center of Cardiology of the Johannes Gutenberg-University Mainz, Germany. Patients meeting the inclusion criteria and none of the exclusion criteria were randomized in a 1:0.75:1 allocation ratio to standard care, app-based physical activity tracking, and app-based physical activity tracking plus coaching, respectively. During randomization, a minimization-based approach^17–19^ was applied to ensure balance between the study arms with regard to age, sex, NT-proBNP, and pre-trial step count.

### Study interventions

All participants received a standardized, semi-structured, counselling session by a specifically trained study physician at Visit 1 with regard to HF guideline recommendations for physical activity.^20^ During the counselling session, participants received general information about physical activity in line with the guidelines, in addition to discussing motivating factors and previously experienced barriers. A booklet including the recommendations was distributed at the end of the session.

Participants from both intervention arms received a fitness wristband (Vívosmart 4, Garmin, USA), which measures the daily step count and provided direct feedback to the patient. Data of the pedometer were automatically transmitted via a Bluetooth connection to a project-tailored, personalized, coaching app, the MyoMobile app, which was installed on a study smartphone (Samsung Galaxy A20e, Samsung, Korea). Only participants randomized to the study arm with activity coaching received personalized app-based coaching for physical activity based upon step count measurements of the fitness wristband via the MyoMobile app. The personalized app-based coaching was based on an algorithm specifically developed with the aim to increase physical activity on a weekly basis using step count as an incentive. The intervention arm with activity coaching was also monitored for compliance with the intervention. Supplementary material online, *Methods* comprises details on the MyoMobile app including screenshots from the app and all motivational messages that were prompted via a pop-up notification depending on the number of steps taken.

### App-based intervention: description of algorithm

Participants in the study arm with physical activity tracking plus app-based coaching were algorithmically instructed to increase their daily number of steps—as calculated from the 5 most active days of the screening phase—by an average of 3000 steps per day over the course of the first 6 weeks of the study. For the remainder of the intervention phase, participants were encouraged to at least maintain or further increase their daily step count. At an average cadence of 100 steps per minute, 3000 steps are equivalent to around 30 min of brisk walking.^21^

**Figure 2:**
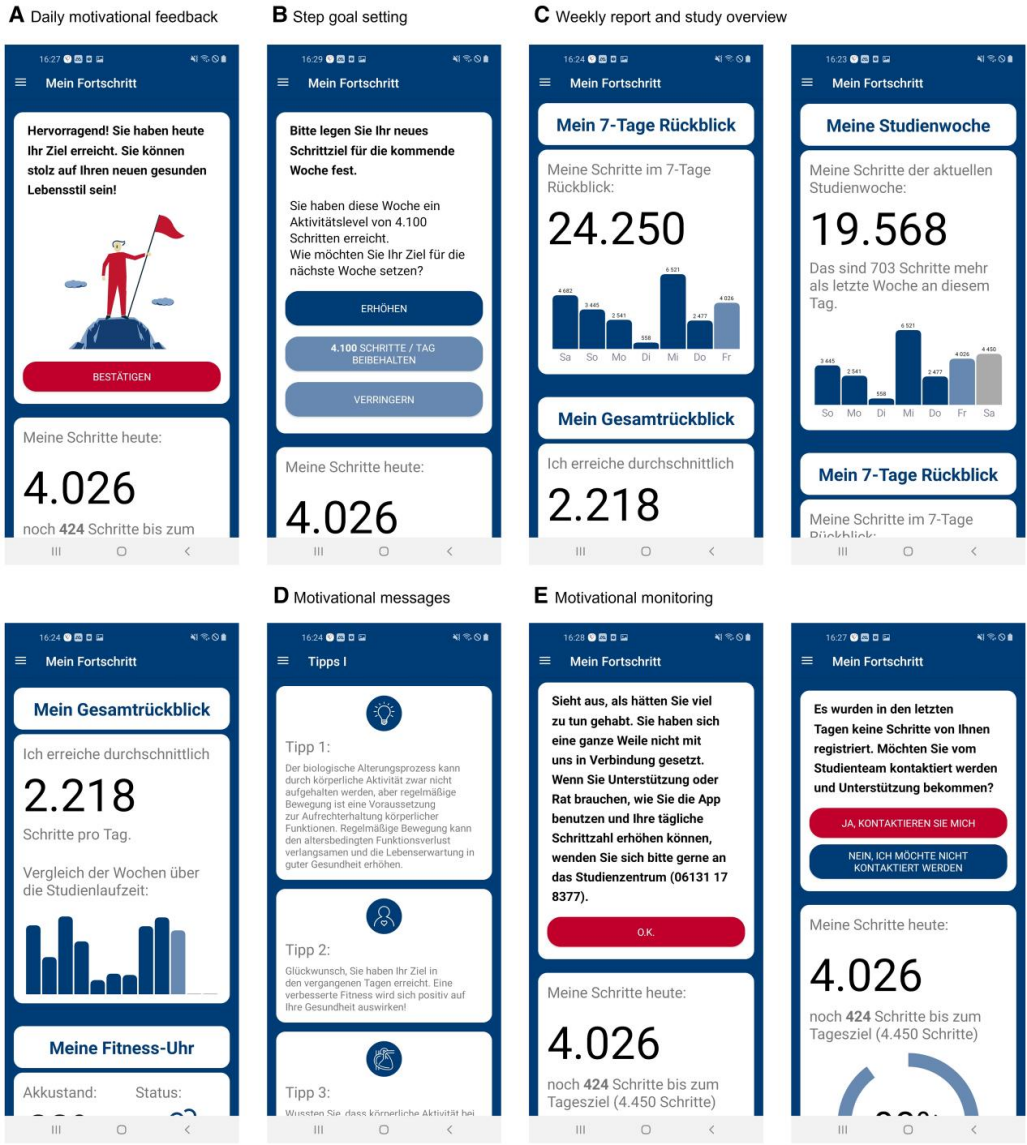
Images of the MyoMobile app. (*A*) Motivational message displaying that the step goal was reached. (*B*) Step goal setting. (*C*) Daily and weekly overview of step count achievements. (*D*) Motivational messages. (*E*) Motivational monitoring.

The app provided coaching by displaying an activity goal (number of steps) and daily feedback via pop-up notifications on the smartphone screen. Participants were reminded daily of their step goal. The daily goal remained the same for one week and the feedback included information on the participants’ daily and weekly achievement by graphical representation (*Figure 2A–C*) as well as pop-up notifications.

**Figure 3:**
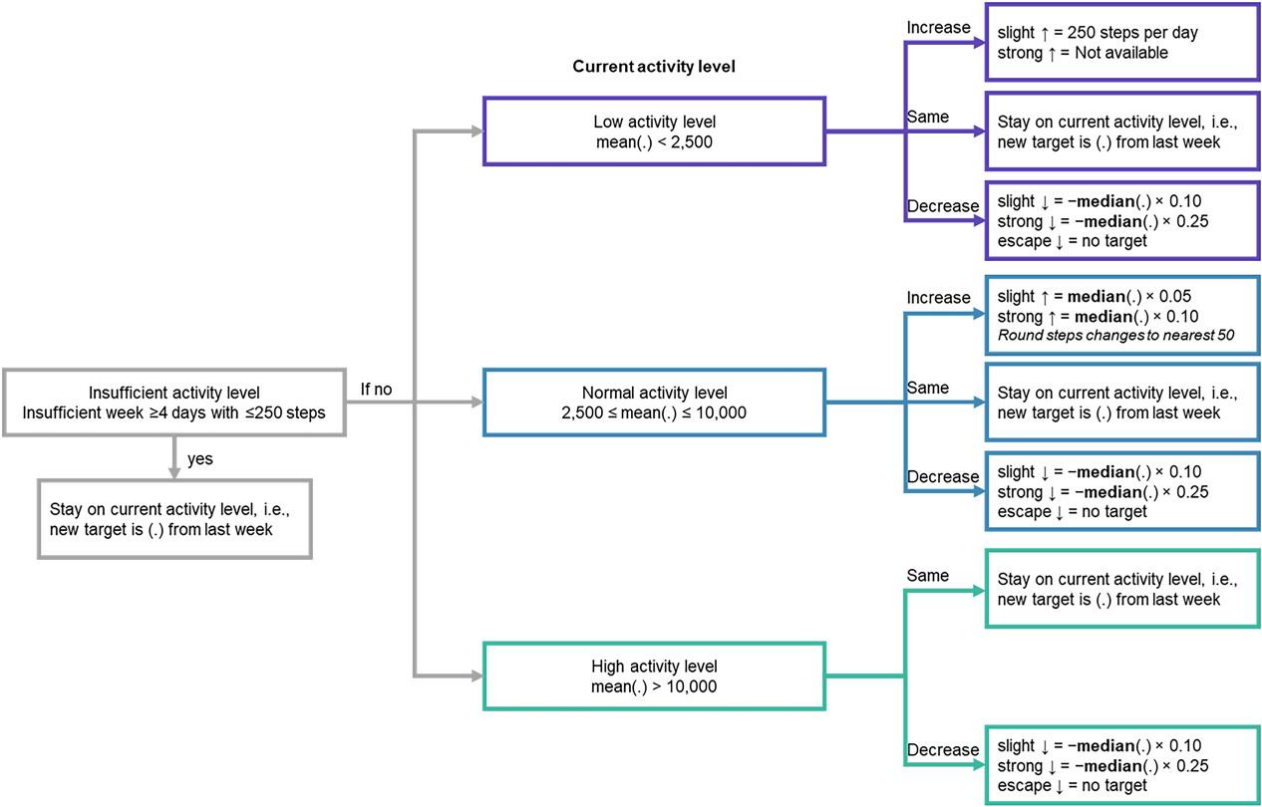
Flowchart of the personalized app-based algorithm developed for the MyoMobile study specifically. The dot in (.) refers to 4 most active days of past week. ↑, increase; ↓, decrease.

Patients’ individual step goals were automatically adjusted once a week based on their step count performance in the preceding week and on their willingness to increase their step goal. The participant indicated this at the end of each study week by filling out a multiple-choice question displayed in the app (see Supplementary material online, *Figure S1A*). At the end of the week, study participants received feedback, including an overview of their performance compared with previous weeks (see Supplementary material online, *Figure S1B*), and a motivational message (see Supplementary material online, *Figure S1C*). The new daily step goal was automatically calculated based on the average step count of the 4 most active days of the previous week. If the average value was higher than the weekly goal (i.e. participants met their target), the participants were asked to further increase their daily step count goal for the subsequent week. The algorithm was inspired by a coaching app developed for an intervention study in Belgium where individuals with chronic obstructive pulmonary disease were coached to increase their physical activity using a smartphone app.^10^ The algorithm is described in more detail in the Supplementary material online, *Methods*. *Figure 3* shows a graphical representation of the algorithm.

### Schedule of activities per visit

**Figure 4:**
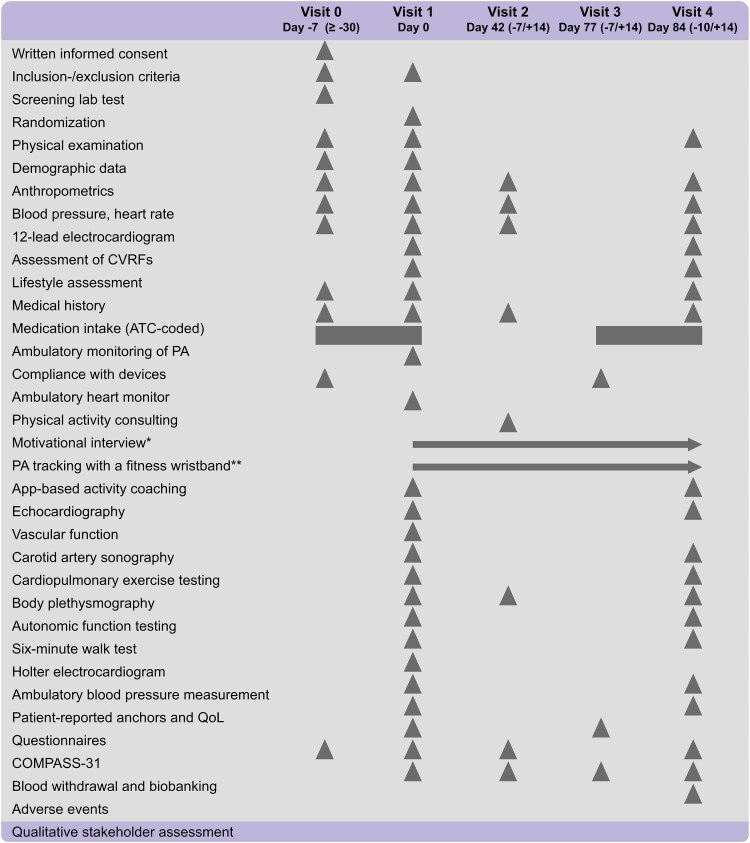
Schedule of study participant activities. Triangle, measure/action to be done at the time point indicated; rectangle, measure/action to be done for a time frame of 7 days; arrow, measure/action to be done continuously, starting from the time point indicated; *only for underperforming participants; **only for participants in intervention group 2; ATC, anatomical therapeutic chemical classification scheme; QoL, quality of life as measured with the Kansas City Cardiomyopathy Questionnaire; PA, physical activity.

The study schedule comprised five visits at the MyoMobile study platform over the entire study period. It consisted of a screening visit (Visit 0), a baseline visit (Visit 1), a visit after 6 weeks of intervention (Visit 2), a visit after 12 weeks of intervention (Visit 3), and a final follow-up visit (Visit 4). *Figure 4* provides a schematic overview of all planned study activities per visit.

Written informed consent was obtained from eligible individuals at the screening visit prior to study enrolment. In addition, study participants received the move monitor (MoveMonitor, McRoberts, The Netherlands) and a heart monitor (T1 Heart Monitor, UMANA, Malta) to record standard one-lead ECG signals. Patients were asked to wear the devices for 7 days, i.e. from Visit 0 to Visit 1. Each patient was screened for inclusion and exclusion criteria at Visit 0 and at Visit 1. At Visit 1 and Visit 4, the results of the move monitor were evaluated regarding wearing time and average daily step count.

Individuals who met the inclusion criteria and none of the exclusion criteria underwent further examinations and deep clinical and molecular phenotyping at Visit 1. Details regarding medication use, cardiovascular risk factors, and disease were gathered through a comprehensive investigational approach, including physical examinations, anthropometric and blood pressure measurements, computer-assisted interviews, and laboratory tests. A comprehensive assessment, encompassing the autonomic nervous system, cardiopulmonary system, functional exercise capacity, and gait, was conducted during the first and fourth visits. These measurements were derived from Holter and 12-lead ECG, cardiopulmonary exercise testing (CPET), and a 6 min walk test utilizing a 3D accelerometer device (MoveTest, McRoberts, the Netherlands). Parameters related to heart rate variability were extracted from 24 h Holter ECG recordings. Additionally, evaluations of cardiac function and structure, such as LVEF and the ratio of early diastolic mitral inflow velocity to early diastolic mitral annulus velocity (E/e′ ratio), were carried out at screening, as well as during the first and fourth visits, using transthoracic echocardiography in accordance with current guidelines.^22^ Questionnaires were administered to participants at both Visit 1 and Visit 4, including assessments of psychological well-being through the Patient Health Questionnaire (PHQ-9) and the Generalized Anxiety Disorder Scale (GAD-7), as well as quality of life via the Kansas City Cardiomyopathy Questionnaire, among others. Refer to the Supplementary material online, *Methods* for further details regarding the various examinations and measurements performed.

Sequential biomaterial sampling was performed to facilitate comprehensive molecular characterizations of HFpEF patients at baseline and after the follow-up period of 12 weeks, enabling insights into the molecular mechanisms underlying observed efficacy and safety effects. High-throughput methods for genotyping, gene expression profiling, and integrative omics analyses will be performed to explore the association between physical activity and changes in biomarkers during the activity coaching as well as various aspects of cardiovascular disease and its risk factors. Blood samples were taken after 8 h of fasting to collect serum, plasma, DNA, and RNA, and midstream urine was collected to investigate renal function and excretion. Blood and urine samples were acquired and stored at −80°C for the establishment of a biobank.

During the second visit, study participants in the study arm with activity coaching who did not meet their step goals received a motivational interview. At Visit 3, the patients received the move monitor and the heart monitor for a second time to wear 7 days, i.e. from Visit 3 to Visit 4. Visit 4 represents the final visit for the participants after 12 weeks of intervention. All assessments conducted at each visit were consistently repeated in the same manner to ensure standardization.

### Safety assessment

Adverse events (AEs) including treatment-related AEs were assessed at each study visit. The onset date of an AE was defined as the date when new signs or symptoms first appeared or an exacerbation of a pre-existing condition occurred. The end date of the AE is defined as the date when the symptoms disappeared or when the event was considered stable by the investigator. The maximum intensity of an AE was rated by the investigator as mild, moderate, or severe. Severe AEs (SAEs) were characterized by substantial impairment of the participant’s normal daily activities. All AEs reported by the patient or identified by the investigator were recorded in a case report form.

### Sample size estimation

No published data are available to estimate the effect of an app-based coaching intervention on physical activity in HFpEF patients. Assumptions for the MyoMobile trial were established based on data from a recent randomized controlled trial that investigated the effect of increasing physical activity levels in patients with chronic obstructive pulmonary disease.^10^ For patients with HFpEF, an age range qualifying for participation similar to that of individuals with chronic obstructive pulmonary disease was assumed.^12^ Based on available data, it was assumed for this study that the minimum difference in daily steps to be identified as statistically significant would be 1400 steps per day over the course of 12 weeks of intervention. With regard to the level of physical activity, a standard deviation in daily steps of 2400 was assumed and a correlation of 0.75 between the baseline and 12-week measurement of the number of daily steps.^10^

A group size of *n* = 62 participants for both the intervention arm with activity coaching and the standard care arm was required for analysis of the primary endpoint using a power of 90% at a two-sided α of 0.05. Assuming a drop-out rate of 25% from baseline to Visit 4, it was assumed that *n* = 83 participants should be randomized to both groups.

To compare the step count between standard care and the intervention arm without activity coaching, a power of 80% at a two-sided α of 0.05 was defined, resulting in a group size of *n* = 47 for this intervention arm. Taking into account the drop-out rate of 25% from baseline to Visit 4, a group size of *n* = 63 was assumed to be randomized to this study arm.

The evaluation of the primary endpoint will be the only confirmatory analysis. The analyses of the secondary and exploratory endpoints will be exploratory in nature without correction for multiple testing.

### Statistical analysis of the primary efficacy endpoint and the secondary and exploratory outcomes

The primary endpoint was defined as the change in average daily step count between the baseline phase (mean of step count data collected with the move monitor during the 7-day period prior to randomization) and the end of the intervention (mean of step count data collected with the move monitor during week 12). The primary efficacy endpoint will be analysed by linear regression analysis with step count at Visit 4 as the dependent variable and the intervention with app-based physical activity tracking plus coaching vs. standard care as the main predictor with adjustment for baseline step count, age, and sex, assuming a two-sided significance level of α = 0.05. The null hypothesis was (H0): ß = 0, i.e. the intervention does not affect the average step count per day after 12 weeks, and the alternative hypothesis was (H1): ß ≠ 0, i.e. the intervention affects the average step count per day after 12 weeks. The primary analysis population is the intention to treat population including all randomized participants with a post-baseline measurement regarding the average step count per day. The secondary and exploratory endpoints will be analysed in a similar manner with linear regressions.

### Pre-specified subgroup analyses of the primary efficacy endpoint

Subgroup analyses of the primary endpoint were pre-specified in the statistical analysis plan and include comparisons between the following groups: women vs. men, BMI < 30 kg/m² vs. BMI ≥ 30 kg/m², LVEF < 50% vs. LVEF ≥ 50%, increase in daily step count of ≥ 1000 vs. <1000 steps, sedentary level vs. non-sedentary level, autonomic dysfunction vs. normal autonomic function, NYHA Class I or II vs. NYHA Class III or IV, beta-blocker intake vs. no beta-blocker intake, a diagnosis of type 2 diabetes mellitus vs. no diagnosis of type 2 diabetes mellitus, history of atrial fibrillation vs. no history of atrial fibrillation, and history of coronary artery disease vs. no history of coronary artery disease. In addition, the following parameters were stratified at the median for subgroup analyses: age, concentration of NT-proBNP, E/e′ ratio, peakVO2, 6 min walk distance, baseline resting heart rate, and heart rate recovery.

### Interim analysis

An interim analysis was planned to re-estimate and adapt the sample size based on conditional power. This analysis was carried out after 50% of participants were allocated and successfully completed study participation (Visit 4). Before conducting the interim analysis of the primary study endpoint, the drop-out rate from baseline (Visit 1) to completion (Visit 4) was assessed, and this criterion was applied: if a drop-out rate of more than 35% is observed, the interim analysis will not be carried out and the trial will continue unchanged. If a drop-out rate of less than 35% is observed, the interim analysis will take place. For the analysis of conditional power for the primary endpoint in the interim analysis, the guiding principles from Mehta and Pocock^23^ for an adaptive increase in sample size were followed. Supplementary material online, *Figure S2* shows the decision zones for the primary (panel A) and main secondary (panel B) endpoint for adapting the sample size.

### Baseline characteristics

Baseline characteristics of the intention-to-treat sample were reported by relative and absolute frequencies for discrete variables, and continuous variables were described by mean and standard deviation, or by median and interquartile range for skewed distributions.

## Results

### Baseline characteristics

The first-patient-first-visit in the MyoMobile trial occurred on 11 November 2020, with the last-patient-last-visit completed on 31 January 2023. *Table 2* reports on the baseline characteristics of the intention-to-treat sample. The sample comprised of *n* = 185 participants with HFpEF. The mean age of the participants was 70.1 ± 8.6 (34.6% women). The mean LVEF was 53.5 ± 7.6. The prevalence of arterial hypertension and dyslipidaemia were 84.3% and 79.5%, respectively. Approximately half of the participants were diagnosed with coronary artery disease (54.6%) or atrial fibrillation (51.9%).

**Table 2:** Baseline characteristics of participants

	Intention-to-treat sample (*N* = 185)
Demographics	
Women, % (*n*)	34.6 (64)
Age (years), mean ± SD	70.1 ± 8.6
Traditional CVRFs, % (*n*)	
Arterial hypertension	84.3 (156)
Dyslipidaemia	79.5 (147)
Diabetes mellitus	35.7 (66)
Obesity	40.5 (75)
Smoking (current)	9.7 (18)
Smoking (former)	42.7 (79)
Comorbidities, % (*n*)	
Atrial fibrillation	51.9 (96)
Chronic kidney disease	19.5 (36)
Chronic obstructive pulmonary disease	21.7 (40)
Coronary artery disease	54.6 (101)
Hx of cancer	20.0 (37)
Hx of myocardial infarction	25.4 (47)
Hx of stroke	7.0 (13)
Hx of venous thromboembolism	12.0 (22)
Liver disease	9.7 (18)
Peripheral artery disease	8.2 (15)
Heart failure status	
NT-proBNP (pg/mL), median (IQR)	418.0 (160.3; 894.3)
LVEF (%), mean ± SD	53.5 ± 7.6
E/e′ ratio, median (IQR)	9.42 (7.36; 11.98)
NYHA functional Class I, % (*n*)	42.7 (79)
NYHA functional Class II, % (*n*)	45.4 (84)
NYHA functional Class III + IV, % (*n*)	11.9 (22)
Medication intake, % (*n*)	
Antidiabetics (A10)	30.3 (56)
Antithrombotic agents (B01A)	91.4 (169)
Cardiac glycosides, antiarrhythmics, and vasodilators (C01)	24.3 (45)
Diuretic agents (C03)	74.1 (137)
Beta blockers (C07)	75.7 (140)
Calcium channel blockers (C08)	22.2 (41)
RAS-acting agents (C09)	84.9 (157)
Lipid-modifying agents (C10)	68.1 (126)
Antidepressants (N06A)	7.0 (13)

CVRFs, cardiovascular risk factors; Hx, history; LVEF, left ventricular ejection fraction; E/e′ ratio, ratio of early diastolic mitral inflow velocity to early diastolic mitral annulus velocity; NYHA, New York Heart Association functional classification; RAS, renin-angiotensin system. Medication intake is noted with the Anatomical Therapeutic Chemical (ATC) code in brackets.

### MyoMobile app

*Figure 2* includes screenshots from the MyoMobile app. Participants from both intervention arms were registered on the MyoMobile app using a QR code generated during randomization (see Supplementary material online, *Figure S3*). The QR code contained the participant’s intervention group, MyoMobile app ID, and average daily step count calculated during the screening period. The step count data from the fitness wristband were updated to the MyoMobile app via Bluetooth to visualize the participant’s step count achievements.

The MyoMobile app visualized the participant’s average step count per day and study week, allowing participants to monitor their achievements. Supplementary material online, *Figure S4* displays screenshots of the app for the intervention arm without app-based coaching. Similarly to the statistics made available to the intervention arm with coaching, these participants were able to monitor their progress per day, the current study week, and the previous study weeks (see Supplementary material online, *Figure S5*).

Every evening and at the end of every study week, the MyoMobile app displayed a notification (see Supplementary material online, *Table S3*) informing the participant whether they achieved their daily step count goal. The feedback was personalized based on the magnitude of the step count increase or decrease. The weekly feedback was based on the mean of the participant’s 4 most active days (their ‘activity level’). The texts for the weekly notifications are described in Supplementary material online, *Table S4*. After receiving the weekly feedback, the participant was asked to set a new step goal. If patients chose to decrease or not increase their step target, or opted for the escape option, they were prompted with a multiple-choice question asking why they made that decision (Supplementary material online, *Table S5*).

Motivational push notifications were sent on Days 3 and 5 in the morning of each study week to encourage participants to increase their physical activity level. The content of the motivational notification depended on whether the step goals on Days 1 and 2 and on Days 3 and 4 were reached. The motivational notifications were based on behavioural change techniques and primarily provided information about health consequences attributable to an increase in the daily step count and tips on how to self-monitor and plan the daily step count. The content of the motivational messages is available and can be requested from the principal investigator. Supplementary material online, *Methods* contain a detailed description of the MyoMobile app, and Supplementary material online, *Figures S6–S9* additional details about the visual layout of the app.

## Discussion

The MyoMobile study was a single-centre, randomized, controlled, three-armed parallel group clinical trial to examine the effect of personalized app-based physical activity coaching intervention compared with standard care on physical activity in patients with HFpEF. The study rationale was based on the poor implementation of physical activity guidelines and by previously research highlighting the benefits of increased physical activity for health in individuals with chronic illnesses. However, to our knowledge, this is the first trial investigating the effect of a personalized coaching app on physical activity in patients with HFpEF.

The MyoMobile trial was conceptualized as an EE2 trial, integrating the principles of efficacy trials designed for regulatory approval with the pragmatic considerations of effectiveness trials aimed at evaluating real-world applicability and practical impact on everyday life. By investigating the impact of a 12-week personalized, app-based activity coaching intervention on daily step count in patients with HFpEF, the MyoMobile trial extends beyond the confines of traditional clinical trial settings. The research platform incorporates a three-armed parallel group design, mirroring the diversity of interventions that might be encountered in routine clinical practice. The inclusion of a broad HFpEF patient population, reflective of those seen in everyday healthcare settings, aligns with the principles of an effectiveness study, where the goal is to assess how well the intervention performs in real-world scenarios.^14^ The trial’s pragmatic approach, combined with comprehensive assessments of standard measures of exercise tolerance (i.e. oxygen uptake and 6 min walk distance), cardiac structure and function, quality of life, autonomic function, molecular profiling, and standard biomarkers, positions MyoMobile as a leading study into the real-world efficacy of app-based coaching for enhancing physical activity levels in HFpEF patients.

A pivotal aspect explored in the MyoMobile trial is the integration of personalized app-based coaching interventions using smartphones and wearables, specifically designed for elderly individuals with HF, making it easier to implement in the normal daily routine at their own pace. Unlike supervised exercise programmes that typically require in-person attendance and structured sessions, the MyoMobile app-based intervention provides greater flexibility. This personalized, self-managed approach may enhance adherence compared to traditional programmes, which often experience declining compliance after 3 months.^9^ The simplicity of an app-based coaching tool, designed specifically for the elderly HFpEF population, may also make it easier to use and sustain over the long term.

The trial delves into both the efficacy and effectiveness of digital tools, while also shedding light on their feasibility and acceptance within the elderly HF population. This exploration extends beyond the conventional realms of clinical trials, offering a nuanced understanding of how elderly individuals in the HF setting engage with digital technology, apps, and smartphones. This approach offers novel insights into the practicality and user-friendliness of digital health interventions among an age group that is often less represented in technology adoption studies, thereby enhancing our understanding of how to effectively integrate these tools into the care and management of elderly HF patients.

Seifert *et al*.^24^ demonstrate that monitoring personal physical activity using mobile tracking technology is particularly effective in the younger elderly male population with a keen interest in technology. In addition, the systematic review conducted by Aslam *et al*.^25^ shows that there are preliminary indications that digital health interventions including motivational approaches might be beneficial for the improvement of physical activity. However, studies that include both app-based motivational coaching and monitoring have not been conducted yet in the elderly population with HFpEF. There is a pressing need for studies that encompass motivational and usability aspects, and the MyoMobile trial addresses exactly this need. In the MyoMobile trial, significant attention is devoted to incorporating motivational messages and adopting a personalized approach to address the participants’ barriers to physical activity.

The MyoMobile trial thus contributes to the evolving discourse on the practicality and efficacy of digital interventions, providing a valuable foundation for future endeavours aiming to leverage technology for the well-being of elderly HF patients. The MyoMobile app’s potential to increase step counts and overall physical activity remains to be demonstrated. If proven effective, it could be used in future research for monitoring physical activity in HFpEF trials. Additionally, the app may have clinical applications, encouraging HFpEF patients to adopt more active lifestyles, and could become a valuable tool in promoting heart-healthy behaviours.

Standard care for HFpEF or HFmrEF patients includes SGLT2 inhibitors,^26^ HF self-care education, exercise, weight loss for obese individuals, and diuretics for overt congestion. However, multiple clinical trials investigating the effect of established pharmacological therapies, such as angiotensin-converting enzyme inhibitors (PEP-CHF^27^), angiotensin receptor blockers (PARAMOUNT,^28^ CHARM-Preserved,^29^ and I-Preserve^30^), spironolactone (Aldo-DHF^31^ and TOPCAT^32^), or angiotensin receptor–neprilysin inhibition (Paragon-HF^33^), have failed to show meaningful improvement of disease symptoms, morbidity, or mortality in patients with HFpEF. Of note, the TOPCAT study highlighted that both poor and intermediate self-reported physical activity levels were linked to an increased risk of HF hospitalization and mortality, after multivariable adjustment for potential confounders.^34^ There is no evidence yet showing that SGLT2 inhibitors improve exercise tolerance in HFpEF patients.^35^ Concerning major depression, a frequent comorbidity of HF, several antidepressant trials failed to achieve improvement of depression or cardiac prognosis.^36^ Exercise training, however, showed promise in reducing depression in HF.^37,38^ But there is a particular lack of studies that address the motivation problem and make broad implementation appear feasible.^38^

Several factors including cardiac impairment, but also peripheral factors such as skeletal muscle deconditioning as well as metabolic impairment in skeletal muscles, are contributors to the advancement of symptoms, such as muscle weakness and exercise limitation, and ultimately worsening of HF.^39^ Skeletal muscle deconditioning in HF leads to activation of muscle ergoreceptors, resulting in increased respiration and sensation of dyspnoea, perception of fatigue, and increased activation of the sympathetic nervous system.^40,41,42^ The poly-etiological nature of HFpEF demands in-depth clinical phenotyping to understand changes in the pathophysiological mechanisms underlying physical activity in this patient group.

Smaller studies^43^ have examined the effect of increased physical activity on biomarkers of HF, such as diastolic function, elevation of NT-proBNP, and cardiac remodelling,^44^ but to date, little effort has been made to discover and investigate pathomechanisms related to physical activity in HFpEF that might be targeted for treatment. Pre-clinical studies using murine models suggest that improved diastolic function resulting from exercise training may be associated with reduced titin-based myocardial stiffness, triggered by decreased *PEVK* phosphorylation and increased *N2B* phosphorylation.^45^ Regarding remodelling of the cardiac extracellular matrix, a large animal model with left ventricular pressure overload showed that, compared with control animals, mRNA levels of *MMP2*, *MMP9*, *TIMP1*, and *TIMP4* normalized after treadmill exercise training, indicating a reduction in cardiac fibrosis.^46^

The deep phenotyping planned for the MyoMobile study, including the establishment of an extensive biobank, will allow investigators to explore clinically relevant translational questions that cannot be addressed to date with the current state of physical activity research in HFpEF. Moreover, the MyoMobile study design allows for the investigation of potential improvements in cardiovascular and cognitive health secondary to increased physical activity, which could provide important insights into the pathophysiology of HFpEF.

Lastly, in managing chronic conditions like HFpEF, a shift towards personalized, holistic healthcare interventions is not only on the horizon but urgently needed. Technological advancements in artificial intelligence, machine learning, and novel devices offer exciting new possibilities. This approach, increasingly embraced by tech-savvy older patients, emphasizes lifestyle modifications alongside traditional pharmacological treatments for a more comprehensive management strategy. Such holistic interventions may not only enhance the effectiveness of pharmacological therapies but also align with emerging value-based reimbursement models, presenting a unified opportunity for healthcare stakeholders to improve patient outcomes. This trend underscores the growing importance of integrating technology with patient-centred care to address the complexities of chronic diseases more effectively.

The MyoMobile trial has several limitations. First, the relatively short 12-week follow-up may not be sufficient to assess the long-term effects of the intervention on clinical outcomes and molecular responses. Second, the minimization-based approach to randomization did not balance the study arms for the distinction between LVEF 41–49% (HFmrEF) and LVEF ≥ 50% (HFpEF), potentially complicating LVEF-stratified analyses due to smaller subgroup sizes. However, similar to the EMPEROR-Preserved trial, which also used a 40% cut-off, the inclusion of patients with HFmrEF offers the opportunity to explore the app’s usefulness for these patients, who experience similar symptoms to those with HFpEF. Lastly, patients with lower digital literacy or limited access to technology may be less likely to benefit from the trial. Efforts to mitigate selection bias included providing participants with smartphones and digital wearables, along with documentation on device usage.

## Conclusions

The MyoMobile study, through its innovative design as a single-centre, randomized, controlled, three-armed, parallel group clinical trial, aims to evaluate the impact of a personalized app-based physical activity coaching intervention on patients with HFpEF. By adopting an EE2 trial model, the study ambitiously bridges the gap between controlled clinical trial conditions and real-world applicability, aiming to generate evidence that spans regulatory approval to practical, everyday impact. Through a comprehensive approach, the trial assesses not only the efficacy and effectiveness of the personalized coaching app but also explores the feasibility and acceptance of digital innovations among the elderly. Exercise intolerance and diminished functional capacity, intricately linked to physical inactivity, constitute key challenges faced by HFpEF patients in their daily lives. While current pharmacological therapies for HFpEF exhibit limited efficacy, the potential for exercise training to modulate physical activity levels offers a promising opportunity for improvement. Personalized app-based physical activity coaching is a promising therapeutic strategy to improve cardiovascular health and disease symptoms in patients with HFpEF.

## Lead author biography


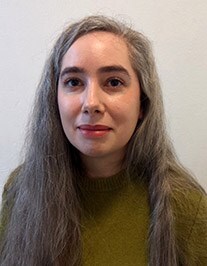


Silav Zeid is a PhD candidate and researcher at the Preventive Cardiology and Medical Prevention at the Center for Cardiology, University Medical Center of the Johannes Gutenberg University Mainz, Germany. Her research focuses on two key areas in HF research: understanding the pathomechanisms underlying autonomic dysfunction in HF and enhancing patient care through digital innovations promoting physical activity within this population. A core aspect of her work is empowering HF patients to increase physical activity, using digital-driven solutions.

## Supplementary Material

ztae096_Supplementary_Data

## Data Availability

The MyoMobile trial constitutes a major scientific effort with high methodological standards and detailed guidelines for analysis and publication. Data are not made available for the scientific community outside the established and controlled workflows and algorithms. To meet the general idea of verification and reproducibility of scientific findings, we offer access to data at the local database in accordance with the ethics vote on request [contact: Prof. Dr Philipp Wild (principal investigator of the MyoMobile trial), myomobile-pk@unimedizin-mainz.de].
