# Supplementary material for: Personalized app-based coaching for improving physical activity in heart failure with preserved ejection fraction patients compared with standard care: rationale and design of the MyoMobile Study
Source: Eur Heart J Digit Health. 2025 Jan 30;6(2):298–309. doi: 10.1093/ehjdh/ztae096 (PMC11914726; doi:10.1093/ehjdh/ztae096)
Supplement: ztae096_Supplementary_Data [file ztae096_supplementary_data.docx]

**SUPPLEMENTAL APPENDIX**

Personalized App-Based Coaching for Improving Physical Activity in HFpEF Patients Compared to Standard Care – Rationale and Design of the MyoMobile Study

Short title: Rationale and Design of the MyoMobile Study

S. Zeid^1,2^ MSc, J.H. Prochaska^1,2,3,4^ MD, A. Schuch^1,2^ MD, S.O. Tröbs^1,2,4^ MD, A. Schulz^1^ PhD,

T. Münzel^5,2^ MD, T. Pies^6^, PhD, W. Dinh^6, 10^ MD, M. Michal^7,2^ MD, P. Simon^8^ MD, PhD,

P.S. Wild^1,3,2,9^ MD, MSc

^1^Preventive Cardiology and Preventive Medicine, Department of Cardiology, University Medical Center of the Johannes Gutenberg University Mainz, Germany;

^2^German Center for Cardiovascular Research (DZHK), partner site Rhine-Main, Mainz, Germany;

^3^Clinical Epidemiology and Systems Medicine, Center for Thrombosis and Hemostasis (CTH), University Medical Center of the Johannes Gutenberg University Mainz, Germany;

^4^Boehringer Ingelheim, Ingelheim am Rhein, Germany;

^5^Department of Cardiology – Cardiology I, University Medical Center of the Johannes Gutenberg University Mainz, Germany.

^6^Bayer AG, Wuppertal, Germany

^7^Department of Psychosomatic Medicine and Psychotherapy, University Medical Center of the Johannes Gutenberg-University Mainz, Germany

^8^Department of Sports Medicine, Rehabilitation and Disease Prevention, Faculty of Social Science, Media and Sport, Johannes Gutenberg-University Mainz, Germany

^9^Systems Medicine Group, Institute of Molecular Biology (IMB), Mainz, Germany

^10^ University of Witten/Herdecke, Witten, Germany; Department of Cardiology, HELIOS Clinic Wuppertal, University Hospital Witten/Herdecke, Wuppertal, Germany

**Address for correspondence**

Philipp Sebastian Wild, MD, MSc

Preventive Cardiology and Preventive Medicine, Department of Cardiology and

Clinical Epidemiology and Systems Medicine, Center for Thrombosis and Hemostasis

University Medical Center of the Johannes Gutenberg-University Mainz

Langenbeckstr. 1, 55131 Mainz, Germany

Phone: +49 6131 17 7163

Email: philipp.wild@unimedizin-mainz.de

**Table of Content**

[**Supplemental Methods** 3](#_Toc178253086)

[**Supplementary Table 1.** Secondary objectives assessing the effects of physical activity between baseline and 12 weeks of intervention as well as between intervention groups 10](#_Toc178253087)

[**Supplementary Table 2.** Tertiary objectives 12](#_Toc178253088)

[**Supplementary Figure 1**. Screenshots from the MyoMobile app for the intervention arm with coaching 15](#_Toc178253089)

[**Supplementary Figure 2.** Decision zones of the interim analysis for the primary and secondary endpoint 16](#_Toc178253090)

[**Supplementary Figure 3.** Screenshots registering on the MyoMobile application 17](#_Toc178253091)

[**Supplementary Figure 4.** Screenshots from the MyoMobile app 18](#_Toc178253092)

[**Supplementary Figure 5.** Step count per week (study week and last 7 days) and per day 19](#_Toc178253093)

[**Supplementary Table 3.** Daily notifications – evening feedback on reached step count 20](#_Toc178253094)

[**Supplementary Figure 6.** Confirmation of whether daily step count goal was reached 21](#_Toc178253095)

[**Supplementary Table 4.** Weekly notification based on the step achievements from day 7 22](#_Toc178253096)

[**Supplementary Figure 7.** Weekly target setting display 23](#_Toc178253097)

[**Supplementary Table 5.** Multiple choice options when the step target was decreased or not increased or when the escape option was chosen 24](#_Toc178253098)

[**Supplementary Figure 8.** Static page on the MyoMobile app with tips and motivational messages 25](#_Toc178253099)

[**Supplementary Figure 9.** Pop-up and push notifications 26](#_Toc178253100)

# **Supplemental Methods**

*Patient examination and assessment of risk factors*

Examination of study participants involved the use of an iE33 echocardiography system with an S5-1 sector array transducer (Philips Healthcare, Hamburg, Germany). Four cardiac cycles, acquired at a mean frame rate of 50/s, were transferred to an image archiving system (Xcelera, Philips Healthcare, Hamburg, Germany) for offline analysis. Left ventricular ejection fraction (LVEF) was calculated using Simpson’s method in the apical four-chamber view. Diastolic inflow peak velocity (E) and peak lateral early diastolic mitral annular velocity (E’) were measured throughout a complete cardiac cycle. All cardiac structure and function measurements adhered to current ASE/EAC recommendations.

Resting heart rate and blood pressure were assessed using an Omron 705-CP electronic oscillometer in a temperature-regulated room, following a five-minute rest period, measured three times at three-minute intervals. Subsequently, anthropometric measurements, including height, weight, and waist- and hip circumference, were conducted.

Vascular function was assessed using three methods at visit 1 and visit 4. Arterial stiffness is measured with the Vascular Explorer, determining parameters such as Pulse Wave Velocity (PWV), Pulse Pressure (PP), Aortic Blood Pressure, and Augmentation Index (AIx), while the Stiffness Index (SI) is measured using PulseTrace. Carotid artery sonography was performed using a Vivid E9 ultrasound system to assess intima-media thickness 1 cm before the carotid bulb, along with screening for plaques in the common, internal, and external carotid arteries. The ankle-brachial index (ABI) was evaluated with a pneumatic cuff and Doppler probe, measuring systolic blood pressure in the A. tibialis posterior and A. dorsalis pedis.

Cardiopulmonary exercise testing (CPET) was conducted on a calibrated electromagnetically-braked cycle ergometer (MasterScreen-CPX, Carefusion, Hoechberg, Germany) using a modified Jones protocol. Tests were continued as symptom-limited in the absence of chest pain, ECG abnormalities, or critical blood pressure changes. Conducted in a temperature-regulated room (23°C to 25°C) with continuous monitoring of ECG, blood pressure, and oxygen saturation, CPET provided determinations of peak aerobic capacity (VO2peak), cardiorespiratory fitness, and heart rate recovery during the post-exercise period. CPET was performed at visits 1 and 4.

Body plethysmography is considered the gold standard for a comprehensive assessment of lung function. Key measures included functional residual capacity (FRCpleth) and specific airway resistance (sRaw), along with total lung capacity (TLC) and residual volume (RV) assessed during deep inspirations and expirations. Airway resistance (Raw), a parameter indicative of airway obstruction, was calculated as the ratio of sRaw to FRCpleth. The parameters measured include vital capacity (VC), forced vital capacity (FVC), FEV1/FVC ratio, peak expiratory flow (PEF), maximal expiratory flow at 75% of FVC (MEF 75), FRCpleth, effective resistance (Reff), diffusing capacity of the lungs for carbon monoxide (DLCO), TLC, and RV. Measurements were conducted using the JAEGR-Pneumotachograph (CareFusion Germany, Höchberg, Germany) at visits 1 and 4.

The VitalScan ANS+ (Medeia Inc, Santa Barbara, California) was used to assess autonomic nervous system (ANS) function by measuring heart rate variability (HRV). The assessment included evaluating the vegetative balance at rest, ANS responses to orthostatic maneuvers, and autonomic responses during Valsalva maneuvers and deep breathing. The VitalScan ANS+ also incorporated cardiac autonomic reflex tests, assessing cardio-vagal innervation through heart rate responses to deep breathing and the Valsalva maneuver, as well as vasomotor adrenergic innervation via blood pressure responses to head-up tilt and Valsalva maneuvers. The ANS test comprised four procedures: a resting phase for baseline HRV and blood pressure, a deep breathing test, a Valsalva test, and a standing or tilt test. Each procedure monitors HRV and beat-to-beat blood pressure, with comparisons made to the baseline. Testing lasted approximately 25 minutes, with automatic interpretive reports generated post-testing. The VitalScan ANS+ was performed at visits 1 (baseline), 2, and 4.

Holter ECG monitoring was conducted using the CardioMem device (Getemed, Teltow, Germany) along with 24-hour blood pressure measurement using the Tonoport V (GE Healthcare, Milwaukee, USA). At the end of visit 1, participants received both devices, with only the Holter ECG provided at follow-up visit 4. Study staff gave participants instructions on how to dismantle the devices during both visits. Holter ECG data were downloaded from the device and imported into Holter ECG analysis software (CardioDay® 2.4.3.16, GETEMED) to extract RR intervals. The RR intervals were then analyzed and filtered for artifacts before being converted into 70 secondary variables, which included time domain, frequency domain, and non-linear indices of HRV using the ‘RHRV’ R package. Patients with active atrial fibrillation, or pacemaker stimulation during the Holter ECG recording time were excluded from the evaluation.

Smokers were categorized based on anamnestic data into smoker (including occasional and permanent smokers) and non-smokers (never and ex-smokers). Active smoking was determined through a computer-assisted personal interview, meeting criteria such as smoking one cigarette per day, at least seven cigarettes per week, one package per month, one cigarillo per day, at least seven cigarillos per week, or two pipes per day.

Obesity was defined as a body mass index (BMI) > 30 kg/m². Type-2 diabetes mellitus was defined by glycated hemoglobin ≥ 6.5%, current use of antidiabetic drugs, or physician diagnosis. Dyslipidemia was defined by a low-density lipoprotein/high-density lipoprotein ratio > 2.5, appropriate medication, triglyceride concentration > 150, or physician-diagnosed dyslipidemia.

Arterial hypertension was assumed if anti-hypertensive drugs were taken or mean systolic blood pressure was ≥ 140 mmHg, or if diastolic blood pressure was ≥ 90 mmHg. A positive family history of myocardial infarction or stroke was defined as a first-degree family member with myocardial infarction or stroke at age > 60 (if male) or > 65 (if female). Chronic kidney disease was defined as an estimated glomerular filtration rate (eGFR) < 60 ml/min/1.73m², using the CKD-EPI formula. Information on venous thromboembolism (VTE) and stroke was collected through a computer-assisted personal interview (CAPI) or medical records. VTE was defined as a past pulmonary artery embolism and/or deep vein thrombosis. Stroke was defined as a prior history of stroke. The prevalence of atrial fibrillation was determined based on physician's diagnosis and self-reporting during the CAPI, as well as findings from resting or Holter ECG assessments. Subsequent classification into atrial fibrillation subtypes - paroxysmal, persistent, and permanent - relied on physician's diagnosis and self-reporting during the CAPI.

Blood samples, obtained from a cubital vein after a fasting period of at least 5 hours, were processed for biobanking and routine blood marker measurement. NT-proBNP levels were measured using a commercially available Elecsys® 2010 proBNP II immunoassay (Roche Diagnostics, Mannheim, Germany).

*MyoMobile App*

Participants from both intervention arms were registered on the MyoMobile app using a QR code generated during randomization (**Supplementary Figure 2**). The QR code contained the participant’s intervention group, MyoMobile app ID, and average daily step count calculated during the screening period. The step count was entered twice into the QR code by the MyoMobile study team for verification purposes. The QR code was scanned on the participant’s smartphone by the MyoMobile study team after the participant was randomized into one of the two intervention groups during the first visit.

Fitness wristband data were recorded and saved every minute for the study arm with app-based physical activity tracking without coaching. The MyoMobile app visualized the participant’s mean step count per day and study week, allowing participants to monitor their achievements (see **Supplementary Figure 3** for screenshots). The data transfer for this intervention arm was not monitored for compliance. Participants were given contact details from the study center so they could reach out should they experience any technical or usage problems.

For the study arm with app-based physical activity tracking plus coaching, the MyoMobile app monitored, recorded, and saved all fitness wristband data per minute. The step count data from the fitness wristband was updated per minute in the MyoMobile app via Bluetooth to visualize the participant’s step count achievements. Similarly to the statistics made available to the intervention arm without coaching, participants were able to monitor their progress per day, the current study week, and the previous study weeks (see **Supplementary Figure 4**). For the intervention arm with activity coaching, future days were displayed in the form of grey bars, which were at the height of the participant’s step count goal. The participant’s current day was visualized with a light blue bar, while past days appeared in the form of a dark blue bar.

Every evening, the MyoMobile app displayed a notification (**Supplementary Table 3**) informing the participants if they did or did not achieve their daily step count goal at 07:30 PM. The daily evening feedback was personalized based on the magnitude of the daily achievement (5% above or below target, more than 5% above target, more than 5% below target, and more than 15% below target. Participants were asked to press ‘okay’ (‘Bestätigen’) when they received this notification (**Supplementary Figure 5**). This notification was used as a proxy for smartphone app interaction.

Participants in the intervention arm with activity coaching received a notification with feedback on their step count at the end of each study week (day 7) at 07:30 PM. The weekly feedback was based on the mean of the participant’s four most active days (their ‘activity level’). The texts for the weekly notifications are described in **Supplementary Table 4**. After receiving the weekly feedback, the participant was asked to set a new step goal (**Supplementary Figure 6**). The goal options presented to the participants, whether to increase, decrease, or stay on the same physical activity level, depended on their activity level compared to their goal in the previous week. If the participant chose to decrease or stay on the same activity level, a screen with a multiple-choice question was prompted to gain insights into the reasons why the participant had chosen to do so (**Supplementary Table 5**).

*Motivational notifications*

The aim of the MyoMobile app was to encourage HFpEF patients to increase their physical activity level. Therefore, motivational push notifications were sent on day three and five at 10:00 AM of each study week. The content of the motivational notification depended on whether the step goals on day 1 and 2, and on day 3 and 4 were reached. The motivational notifications were based on behavioral change techniques and provided primarily information about health consequences attributable to an increase in the daily step count and tips on how to self-monitor and plan the daily step count. An overview of all motivational notifications can be requested from the principal investigator. The motivational notifications were designed in collaboration with the Institute of Sport Science and the Department of Psychosomatic Medicine and Psychotherapy, Johannes Gutenberg University, Mainz, Germany. In general, when participants reached their daily targets, they were displayed notifications about health consequences of physical activity. When participants did not increase or reach their target, the notifications were mainly about action planning, self-monitoring, and overcoming barriers. The notifications were implemented in such a way that the participant always received a new motivational notification (i.e., no overlap in displayed notifications). All motivational messages were additionally accessible on a static page on the MyoMobile app (**Supplementary Figure 7**). When the participant touched the motivational push notification, he or she was redirected to the static page with all motivational messages.

*Monitoring and motivational support in the intervention arm with app-based physical activity tracking plus coaching*

To monitor participant compliance with the intervention, four notifications were forwarded to the study center. A notification was dispatched to the study center under the following circumstances: (1) when the participant failed to record any steps for three consecutive days; (2) when daily app interaction was missed for three consecutive weeks; (3) when the weekly step count fell below 50% of the screening or the previous week’s target within the initial five weeks, i.e., the average weekly percentage of step count reached over this period; and (4) when the participant selected a new weekly target (a slight or significant increase, a slight or significant decrease, maintaining the goal, or choosing the escape option [no target or notifications]).

The first three notifications were reviewed and assessed by the MyoMobile study team, and if necessary, the team reached out to participants requiring additional motivational support. The notifications sent to the study center served as supplementary information for the motivational interview at visit 2. Subsequently, the study team initiated contact by phone.

Individuals who had achieved less than 50% of their target step count over the first five weeks or had missed interacting with the app for three weeks or longer within those initial five weeks were invited to participate in a motivational interview. This interview occurred after six weeks, marking the midpoint of the 12-week intervention, when study participants visited the study center for further biobanking. The MyoMobile study team had received training in motivational interviewing from the Department of Psychosomatic Medicine and Psychotherapy at Johannes Gutenberg University. Motivational interviewing served as a tool to help participants overcome barriers to become more physically active and instigate behavioral changes beneficial to their activity levels.

The first two notifications to the study center also triggered a push notification to the participant’s smartphone. In case of any technical or usage issues, the participant was encouraged to reach out to the study center. If the participant had not recorded any steps for three consecutive days, a push notification was sent, asking if the participant wished to be contacted by a member of the study team (**Supplementary Figure 8A**). When daily MyoMobile application interaction was missed for three consecutive weeks, the participant received a push notification containing the contact details of the study center, encouraging them to reach out when necessary (**Supplementary Figure 8B**).

*MyoMobile step count target algorithm*

Participants from the arm with app-based physical activity tracking plus coaching, who had reached their weekly target and maintained an activity level (mean of four most active days in the previous week) between 2,500 and 10,000, were offered to slightly (5% of median) or strongly (10% of median) increase their target for the coming week (median of four most active days in the previous week). However, participants had the option not to change their activity level (i.e., target remained on the same activity level) or slightly (10%) or strongly (25%) decrease their weekly target or choose the escape option (i.e., no target). The same six options were offered when the target was not reached. The minimum and maximum increase in steps for the participant’s weekly goal were 250 and 1,000 steps per day. If the median activity level was below 2,500 steps, only a fixed increase of 250 steps was offered. If the median activity level was equal to or above 10,000 steps, no further increase was offered, but only a slight or strong decrease, staying on the same activity level, and the escape option. If participants had an activity level of 250 steps or less per day for four days or more, this was recognized as an insufficient activity level. In this case, only the option to stay on the same activity level as the previous week was offered.

# **Supplementary Table 1.** Secondary objectives assessing the effects of physical activity between baseline and 12 weeks of intervention as well as between intervention groups

| 1. Difference in E/E' ratio (change from baseline to 12-week follow-up) [ Time Frame: 12 weeks ]Difference in E/E' ratio (change from baseline (V1) to 12-week follow-up (V4)) |
| --- |
| 1. Difference in left ventricular ejection fraction (LVEF) from baseline to 12-week follow-up (V4) [ Time Frame: 12 weeks ]Difference in LVEF (systolic function) from baseline to 12-week follow-up |
| 1. Difference in quality of life (change from baseline to 12-week follow-up) [ Time Frame: 12 weeks ]Difference in quality of life from baseline to 12-week follow-up (measured with The Kansas City Cardiomyopathy Questionnaire (KCCQ)) |
| 1. Difference in heart rate variability (HRV) (change from baseline to 12-week follow-up) [ Time Frame: 12 weeks ]Difference in HRV from baseline to 12-week follow-up (measured with 24-hour Holter ECG) |
| 1. Difference in peak VO2 (change from baseline to 12-week follow-up) [ Time Frame: 12 weeks ]Difference in peak VO2 from baseline to 12-week follow-up (cardiopulmonary exercise testing) |
| 1. Change in daily non-sedentary daytime activity from baseline to 12-week follow-up [ Time Frame: 12 weeks ]Change in daily non-sedentary daytime activity from baseline to 12-week follow-up (composite measure of movement and locomotion as measured by the Dynaport MoveMonitor) (V4) |
| 1. Difference in gait speed (change from baseline to 12-week follow-up) [ Time Frame: 12 weeks ]Change in gait speed from baseline to 12-week follow-up |
| 1. Difference in NT-proBNP from baseline to 12-week follow-up [ Time Frame: 12 weeks ]Difference in the serum concentration of N-terminal brain natriuretic peptide (NT-proBNP) from baseline to 12-week follow-up |
| 1. Difference in FEV1 (change from baseline to 12-week follow-up) [ Time Frame: 12 weeks ]Difference in forced expiratory volume in one second (FEV1) from baseline to 12-week follow-up |
| 1. Difference in the augmentation index (change from baseline to 12-week follow-up) [ Time Frame: 12 weeks] Difference in the augmentation index from baseline to 12-week follow-up. The augmentation index is an indicator of arterial stiffness; higher values indicate a worse outcome |
| 1. Correlations of gait speed [ Time Frame: 12 weeks ]Correlations of gait speed during an intermittent supervised test to data assessed in patients' home environment |
| 1. Difference in METs (change from baseline to 12-week follow-up) [ Time Frame: 12 weeks ]Change in metabolic equivalents (METs) from baseline to 12-week follow-up |
| 1. Difference in daily step count between the intervention groups (change from baseline to 12-week follow-up) [ Time Frame: 12 weeks ]Difference in daily step count from baseline to end of study (comparing the two intervention groups only) |

# **Supplementary Table 2.** Tertiary objectives

| 1. Difference in biomarkers of autonomic function (change from baseline to 6-week follow-up) [ Time Frame: 6 weeks ] |
| --- |
| 1. Difference in biomarkers of autonomic function (change from baseline to 12-week follow-up) [ Time Frame: 12 weeks ] |
| 1. Difference in biomarkers of heart failure (change from baseline to 6-week follow-up) [ Time Frame: 6 weeks ] |
| 1. Difference in biomarkers of heart failure (change from baseline to 12-week follow-up) [ Time Frame: 12 weeks ] |
| 1. Difference in biomarkers of cardiovascular disease (change from baseline to 6-week follow-up) [ Time Frame: 6 weeks ] |
| 1. Difference in biomarkers of cardiovascular disease (change from baseline to 12-week follow-up) [ Time Frame: 12 weeks ] |
| 1. Difference in biomarkers of metabolic diseases (change from baseline to 6-week follow-up) [ Time Frame: 6 weeks ] |
| 1. Difference in biomarkers of metabolic diseases (change from baseline to 12-week follow-up) [ Time Frame: 12 weeks ] |
| 1. Difference in biomarkers of renal diseases (change from baseline to 6-week follow-up) [ Time Frame: 6 weeks ] |
| 1. Difference in biomarkers of renal diseases (change from baseline to 12-week follow-up) [ Time Frame: 12 weeks ] |
| 1. Difference in biomarkers of cancer (change from baseline to 6-week follow-up) [ Time Frame: 6 weeks ] |
| 1. Difference in biomarkers of cancer (change from baseline to 12-week follow-up) [ Time Frame: 12 weeks ] |
| 1. Difference in biomarkers of pulmonary diseases (change from baseline to 6-week follow-up) [ Time Frame: 6 weeks ] |
| 1. Difference in biomarkers of pulmonary diseases (change from baseline to 12-week follow-up) [ Time Frame: 12 weeks ] |
| 1. Difference in biomarkers of inflammation (change from baseline to 6-week follow-up) [ Time Frame: 6 weeks ] |
| 1. Difference in biomarkers of inflammation (change from baseline to 12-week follow-up) [ Time Frame: 12 weeks ] |
| 1. Difference in biomarkers of immunity (change from baseline to 6-week follow-up) [ Time Frame: 6 weeks ] |
| 1. Difference in biomarkers of immunity (change from baseline to 12-week follow-up) [ Time Frame: 12 weeks ] |
| 1. Difference in biomarkers of oxidative stress (change from baseline to 6-week follow-up) [ Time Frame: 6 weeks ] |
| 1. Difference in biomarkers of oxidative stress (change from baseline to 12-week follow-up) [ Time Frame: 12 weeks ] |
| 1. Difference in biomarkers of hypercoagulability (change from baseline to 6-week follow-up) [ Time Frame: 6 weeks ] |
| 1. Difference in biomarkers of hypercoagulability (change from baseline to 12-week follow-up) [ Time Frame: 12 weeks ] |
| 1. Difference in biomarkers of vascular/endothelial function (change from baseline to 6-week follow-up) [ Time Frame: 6 weeks ] |
| 1. Difference in biomarkers of vascular/endothelial function (change from baseline to 12-week follow-up) [ Time Frame: 12 weeks ] |
| 1. Difference in biomarkers of carotid atherosclerosis (change from baseline to 6-week follow-up) [ Time Frame: 6 weeks ] |
| 1. Difference in biomarkers of carotid atherosclerosis (change from baseline to 12-week follow-up) [ Time Frame: 12 weeks ] |
| 1. Difference in biomarkers of methylation (change from baseline to 6-week follow-up) [ Time Frame: 6 weeks ] |
| 1. Difference in biomarkers of methylation (change from baseline to 12-week follow-up) [ Time Frame: 12 weeks ] |
| 1. Difference in anthropometrics (change from baseline to 6-week follow-up) [ Time Frame: 6 weeks ] |
| 1. Difference in anthropometrics (change from baseline to 12-week follow-up) [ Time Frame: 12 weeks ] |
| 1. Difference in biomarkers of psychosomatic diseases (change from baseline to 12-week follow-up) [ Time Frame: 12 weeks ] |
| 1. Difference in biomarkers of physical activity [ Time Frame: 12 weeks ] |
| 1. Difference in biomarkers of sedentary daytime activities [ Time Frame: 12 weeks ] |
| 1. Differences in accelerometry [ Time Frame: 12 weeks ] |
| 1. Evaluation of compliance of study participants with the mobile devices [ Time Frame: 12 weeks ] |
| 1. Evaluation of functionality of the mobile devices [ Time Frame: 12 weeks ] |
| 1. Evaluation of reliability of the mobile devices [ Time Frame: 12 weeks ] |
| 1. Explorative evaluation of reliability of mobile device measurements (e.g. by comparing systolic blood pressure measurements between mobile devices and routine measurements) |

# **Supplementary Figure 1**. Screenshots from the MyoMobile app for the intervention arm with coaching


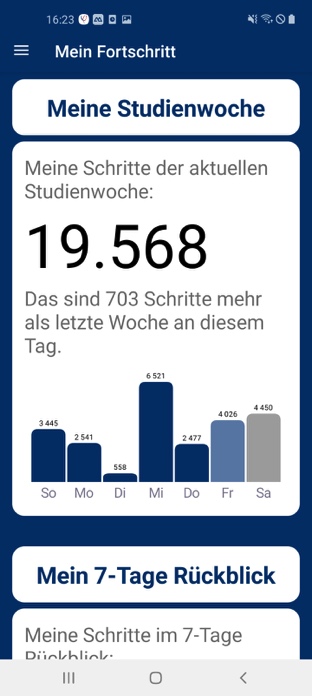


Panel B. My study week


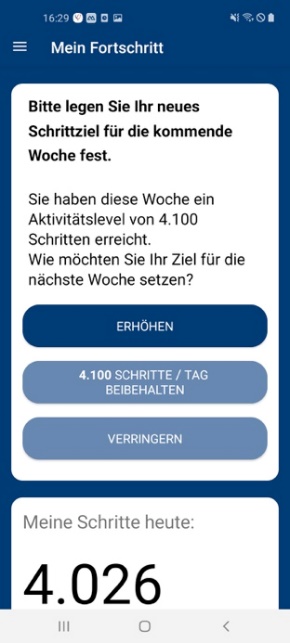

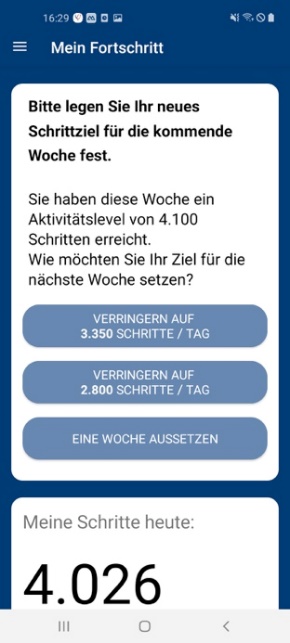


Panel A. Display of the weekly goal setting


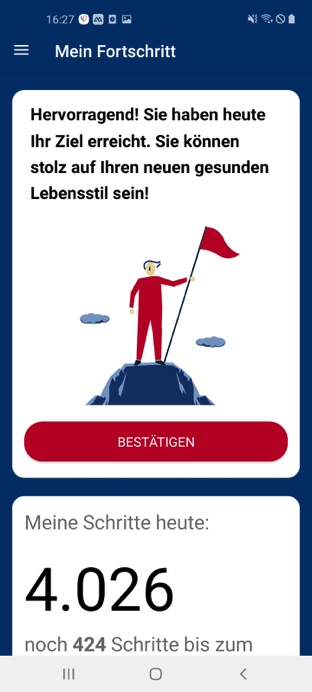


Panel C. Step goal reached

Panel A. Once a week, participants from the intervention arm with coaching were asked to set a new step goal. Participants can choose to increase their number of steps, set the goal at their current step level, or decrease their step goal. Panel B. Step count of the current study week; Panel C. Motivational message displaying that the step goal was reached.

#
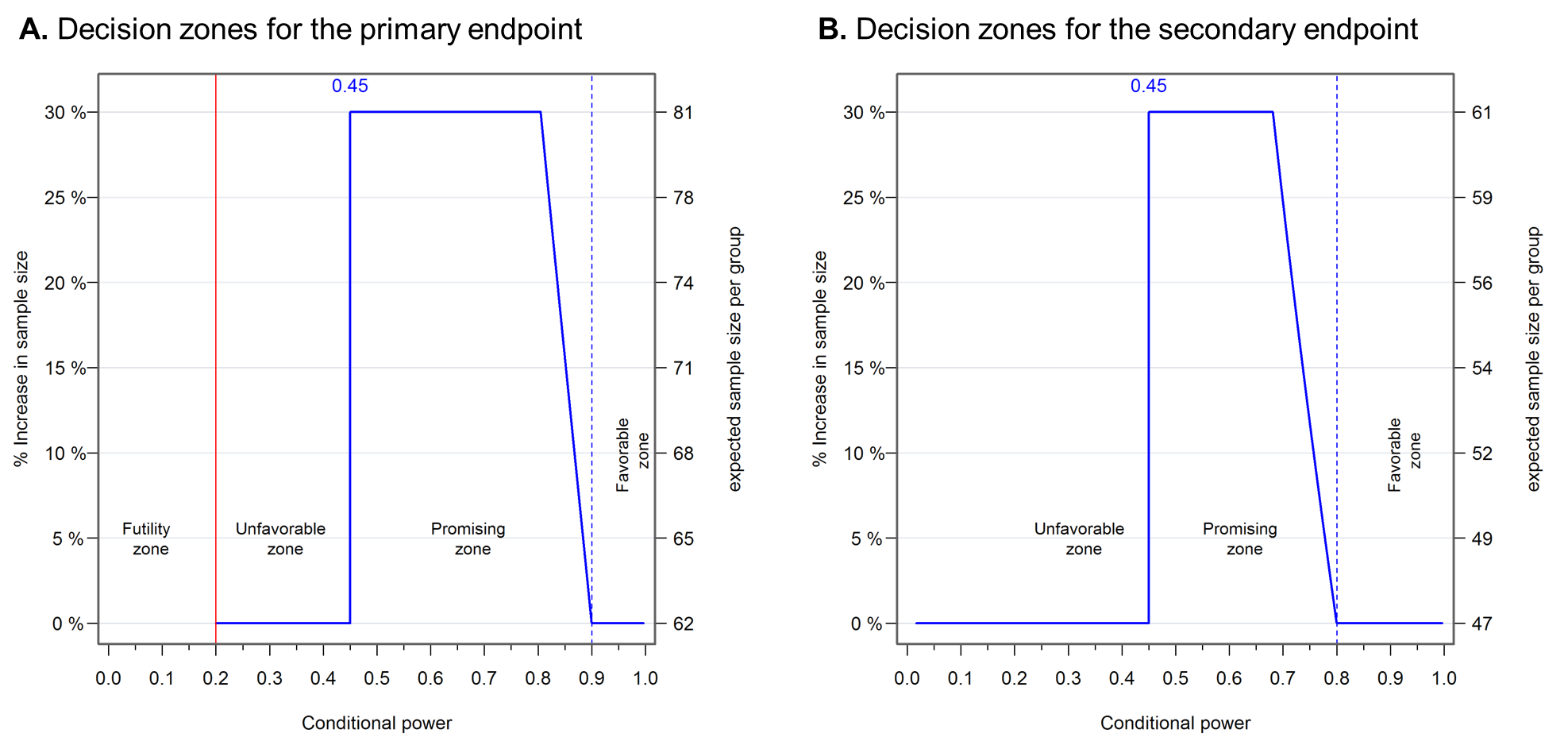
**Supplementary Figure 2.** Decision zones of the interim analysis for the primary and secondary endpoint

# **Supplementary Figure 3.** Screenshots registering on the MyoMobile application

# **Supplementary Figure 4.** Screenshots from the MyoMobile app

# **Supplementary Figure 5.** Step count per week (study week and last 7 days) and per day

# **Supplementary Table 3.** Daily notifications – evening feedback on reached step count

| Difference to daily goal | Notification |
| --- | --- |
| **5% above or below target** | Gut gemacht! Sie haben heute Ihr Ziel erreicht. Weiter so! |
| **More than 5% above target** | Hervorragend! Sie haben heute Ihr Ziel erreicht. Sie können stolz auf Ihren neuen gesunden Lebensstil sein! |
| **More than 5% below target** | Gut gemacht! Ihr Ziel heute war zum Greifen nahe. Bleiben Sie dran! |
| **More than 15% below target** | Heute haben Sie Ihr Ziel leider nicht erreicht. Das kann ab und zu passieren! Achten Sie auf Ihre Schritte während des Tages und Sie werden Ihr Wochenziel erreichen! |

# **Supplementary Figure 6.** Confirmation of whether daily step count goal was reached

# **Supplementary Table 4.** Weekly notification based on the step achievements from day 7

| Difference to weekly goal | Notification |
| --- | --- |
| **More than 5% above target** | Hervorragend! Sie haben diese Woche Ihr Ziel erreicht. Sie können stolz auf Ihren neuen gesunden Lebensstil sein! |
| **5 to 15% below step target** | Gut gemacht! Ihr Ziel war diese Woche zum Greifen nahe. Bleiben Sie dran! |
| **15% or more below step target** | Diese Woche haben Sie ihr Ziel leider nicht erreicht. Das kann passieren. Achten Sie auf Ihre Schritte und Sie werden Ihr Ziel nächste Woche erreichen! |

# **Supplementary Figure 7.** Weekly target setting display

# **Supplementary Table 5.** Multiple choice options when the step target was decreased or not increased or when the escape option was chosen

| Multiple choice options |
| --- |
| 1. Mir fällt es schwer, mich zu motivieren. |
| 2. Aufgrund ärztlicher Empfehlung soll ich mich schonen. |
| 3. Ich mache mir Sorgen meiner Gesundheit durch die Bewegung zu schaden. |
| 4. Im Moment habe ich persönliche Probleme, die mich von mehr Bewegung abhalten. |
| 5. Ich fühle mich gestresst, meine Tages- und/oder Wochenziele zu erreichen. |
| 6. Andere. |

# **Supplementary Figure 8.** Static page on the MyoMobile app with tips and motivational messages

# **Supplementary Figure 9.** Pop-up and push notifications
